# Coronal Alignment Does Not Adequately Predict Femoral Rotation Axes in Total Knee Arthroplasty: Application of a 3D Image-Based Robotic-Assisted Arthroplasty Platform

**DOI:** 10.3390/bioengineering12070727

**Published:** 2025-07-01

**Authors:** Utkarsh Anil, Catherine Di Gangi, Lachlan Anderson, Charles C. Lin, Matthew Hepinstall, Morteza Meftah, Armin Arshi

**Affiliations:** 1 Department of Orthopedic Surgery, NYU Langone Orthopedic Hospital, New York, NY 10010, USA; catherine.digangi@nyulangone.org (C.D.G.); matthew.hepinstall@nyulangone.org (M.H.); morteza.meftah@nyulangone.org (M.M.); armin.arshi@nyulangone.org (A.A.); 2 Anne Burnett Marion School of Medicine at TCU, Fort Worth, TX 76104, USA; l.f.anderson@tcu.edu

**Keywords:** posterior condylar angle, transepicondylar axis, gap balancing, robotic CT planning, patient-specific instrumentation, three-dimensional CT imaging

## Abstract

(1) Introduction: Precise femoral component rotation is critical for achieving symmetric flexion-gap balance and physiologic patellofemoral tracking in mechanically aligned total knee arthroplasty (TKA). Surgeons often infer an appropriate rotational target from the patient’s coronal limb alignment, yet the strength of this relationship remains uncertain. (2) Methods: We identified 695 consecutive patients undergoing primary TKA with a preoperative planning CT scan. The surgical transepicondylar axis (sTEA) and posterior condylar axis (PCAxis) were identified and the angle between them was measured. The angle between the mechanical axis of the femur and tibia was used to measure the coronal alignment of the limb. (3) Results: The mean sTEA was 3.0° externally rotated to the PCAxis (range 3.1° internal to 9.2° external). The mean coronal alignment was 4.3° varus (range −12.5° valgus to 24.5° varus). There were 465 patients with >2° varus and 101 patients with >2° valgus. The mean sTEA was 2.9 ± 1.9° externally rotated relative to the PCAxis in the valgus group and 2.8 ± 2.0° in the varus group, with no statistically significant difference (*p* = 0.7). (4) Conclusions: There is significant variation in the femoral rotation axes between patients, but no significant relationship between overall limb coronal alignment and the magnitude of femoral rotation axes variation. This reinforces the need for independent assessment of rotational landmarks when performing mechanically aligned TKA.

## 1. Introduction

Rotational alignment of the femoral component in total knee arthroplasty (TKA) is fundamental in producing optimal medial-lateral soft tissue balance through the optimization of balanced flexion gaps, stable collateral ligaments, and satisfactory patellofemoral mechanics to prevent maltracking [1,2]. While many patient-specific anatomical relationships can influence this alignment, osseus coronal alignment is likely one of the most important that has yet to be fully characterized. Coronal alignment has been previously shown to affect the location of the trochlear groove and the geometry of the distal femoral trochlea, both of which impact patellar kinematics at various stages in flexion [3,4,5]. Additionally, coronal alignment may affect the rotational alignment and soft tissue balance through its influence on the Q-angle, defined as the angle between the line through the center of the patella to the anterior superior iliac spine and the line between the tibial tubercle through the center of the patella [6]. Due to this effect, understanding the relationship between femoral rotation and coronal alignment can help surgeons predict soft tissue balance between flexion and extension, improving functional mechanics and clinical outcomes.

Different techniques have been employed to attain appropriate femoral alignment, with one of the most common being landmark-based measured resection. Surgeons employing measured resection set femoral component rotation using bony landmarks, including Whiteside’s line, the surgical transepicondylar axis (sTEA), and/or the posterior condylar axis (PCAxis). Whiteside’s line refers to an anteroposterior axis of the femur, extending from the deepest portion of the trochlear groove to the center of the intercondylar notch. The sTEA is defined as the line connecting the sulcus in the medial epicondyle and prominence of the lateral epicondyle of the femur. It connects the approximate origins of the collateral ligaments and is typically found roughly perpendicular to Whiteside’s line [7,8,9]. In practice, the sTEA is commonly approximated to be 2–3 degrees externally rotated to the PCAxis, defined as a line tangent to the posterior aspects of the femoral condyles, but this relationship is acknowledged to be variable. While variable and surgeon dependent, these two axes remain the most widely used within contemporary mechanically aligned TKA instrumentation and were thus explicitly explored in this study.

Surgeons targeting “anatomic” or “unrestricted kinematic” alignment typically set the femoral component rotation parallel to the PCAxis, which creates a reasonably balanced flexion gap if the tibia is cut anatomically. In the mechanical alignment paradigm, landmark-based methods typically set the femoral component perpendicular to Whiteside’s line, parallel to the palpated approximation of the surgical transepicondylar axis (sTEA), or 3 degrees externally rotated to the PCAxis [10]. Relationships between the landmarks vary between knees, however, making ideal rotational targets and landmarks less clear.

One established practice is to select 3 degrees of external rotation to the PCAxis in varus knees and 5 degrees of external rotation to the PCAxis in valgus knees. Traditional teaching notes that TKA in valgus knees can require greater than 3 degrees of external rotation relative to the PCAxis but acknowledges that some varus knees may similarly require additional external rotation as well, arguing that extreme outliers in constitutional varus and/or valgus alignment reflect differences in rotation [10,11,12]. Thus, it is unclear whether coronal limb alignment predicts optimal femoral component rotation and whether there is significant population-level variation.

Previous studies have attempted to characterize this relationship using imaging modalities such as MRI and CT, reasoning that an externally rotated sTEA may compensate for valgus limbs and vice versa. These studies were, however, in smaller patient cohorts, excluded severe deformities, and used different types of imaging modalities [13,14,15,16]. Whether the findings would persist in a larger, more heterogeneous patient population remains unknown.

The purpose of this study is to understand the morphology of the femoral rotation axes and their relationship with overall limb coronal alignment. We aim to examine variability in the posterior condylar angle (PCAngle) between the sTEA and the PCAxis across a large cohort of arthritic knees and describe how this variability associates with coronal limb alignment. This relationship has secondary implications for surgical planning in TKA, as surgeons frequently rely on coronal alignment to infer rotational targets. Clarifying whether a reliable correlation exists may help improve flexion gap balancing, particularly when intraoperative landmark visualization is limited.

## 2. Materials and Methods

### 2.1. Study Population

This study received institutional review board approval. Between 12 April 2023 and 20 August 2024, a total of 695 patients who underwent robotic arm-assisted unilateral primary TKA at a large academic medical center were retrospectively identified. All cases were performed using the Mako robot, which utilizes 3-dimensional planning based on preoperative computed tomography (CT) scans (v2.0; Stryker, Kalamazoo, MI, USA). Patients with retained implants from fracture, ligament reconstruction, osteotomy fixation, or prior partial knee arthroplasty were excluded from this analysis. Conversions and revision cases were further excluded.

### 2.2. Data Collection

Patient demographic variables, including age, sex, and race, were collected from the electronic medical record (EMR) system (Epic Caboodle. version 15; Verona, MI, USA) using Microsoft SQL Server Management Studio 2017 (Redmond, WA, USA). Other variables collected from the EMR included body mass index (BMI), American Society of Anesthesiologists (ASA) score, and Charlson Comorbidity Index (CCI). A summary of baseline demographic variables in the complete cohort is presented in Table 1. Intraoperative data was collected from screen captures, including overall coronal limb alignment and PCAxis to sTEA rotation.

The overall coronal limb alignment was measured intraoperatively using resting limb position after initial osteophyte removal, without correcting for varus or valgus angulation. There was no correction for resting flexion contracture during measurement.

**Figure 1:**
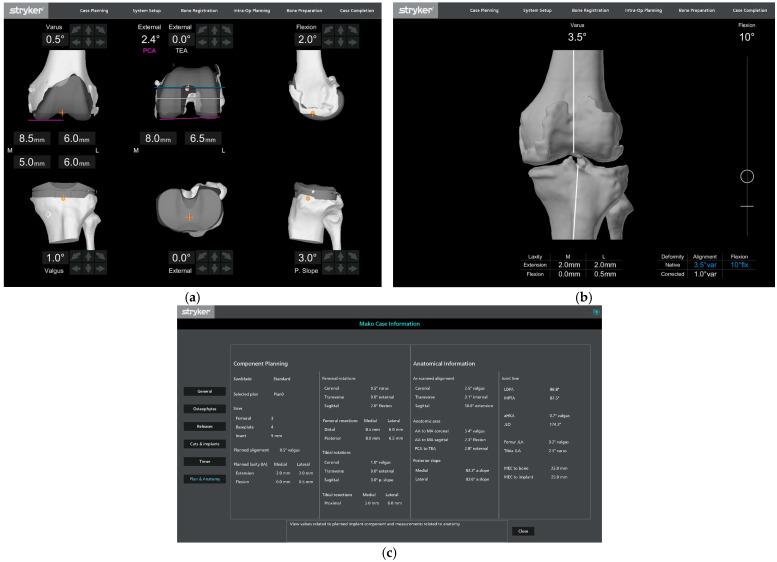
(**a**) Robotic screen captures. (**b**) Implant planning. (**c**) Initial assessment. Case summary anatomical information section. Note: This example of a left knee demonstrates a sTEA to PCAxis of 2.4 degrees external rotation (PCAngle = +2.4 deg).

The PCAxis was defined as a line tangent to the most posterior points of the lateral and medial femoral condyles. The sTEA was defined as the line connecting the deepest portion of the middle sulcus of the medial epicondyle and the lateral epicondylar prominence [17] (Figure 1).

PCAngle was defined as the rotation of the sTEA to the PCAxis, with external rotation reported with positive values and internal rotation reported with negative values. Varus limb alignment was reported with positive values, and valgus limb alignment was reported with negative values. Coronal alignments greater than 2° varus were categorized as varus and those greater than 2° valgus were categorized as valgus; the remaining knees were considered to have neutral alignment.

### 2.3. Data Analyses

All statistical analyses were performed using R (v4.1.2; R Core Team 2021). All continuous variables are reported as means and medians with their ranges and compared using the Wilcoxon rank sum test. Categorical variables were compared using Fisher’s exact test. Correlation between femoral rotation morphology and coronal limb alignment was calculated using Pearson’s product–moment correlation test.

## 3. Results

### 3.1. Femoral Rotation Morphology

**Figure 2:**
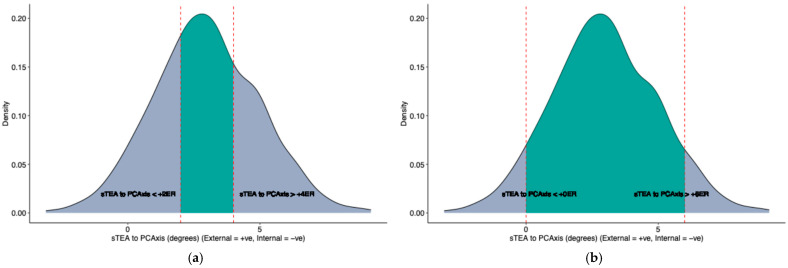
Surgical transepicondylar axis (sTEA) to Posterior condylar axis (PCAxis) density distribution. (**a**) The shaded area represents patients in whom the sTEA was between 2 and 4 degrees externally rotated relative to the PCAxis. (**b**) The shaded area represents patients in whom the sTEA was between 0 and 6 degrees externally rotated relative to the PCAxis.

In the present study, the mean PCAngle between sTEA to PCAxis was +3.0° external rotation (range of −3.1° internal rotation to +9.2° external rotation). In 39% of patients (270 patients from 695), the relative rotation of sTEA to PCAxis was between 2° and 4° external rotation. In 67% of patients (466 patients from 695), the relative rotation of sTEA to PCAxis was 1° to 5° external rotation (Table 2, Table S1). In 93% of patients (647 patients from 695), the relative rotation of sTEA to PCAxis was greater than 0° (external rotation), and in 6.2% of patients (43 patients from 695), the relative rotation of sTEA to PCAxis was less than 0° (internal rotation). The density distribution of PCAngle values is presented in Figure 2.

### 3.2. Overall Corona Limb Alignment

The mean coronal alignment in this analysis was +4.3° varus (range −12.5° valgus to +24.5° varus). Coronal limb alignment was more than 2° varus in 67% of patients (465 of 695) and more than 2° valgus in 15% of patients (101 of 695). (Table 3). There were statistically significant differences between the varus and valgus groups in sex (84% vs. 65% Female, *p* = < 0.001) and BMI (30.1 ± 5.39 vs. 31.8 ± 4.91, *p* = 0.004). There were no statistically significant differences between the varus and valgus groups in age (67.4 ± 10.31 vs. 67.9 ± 8.91, *p* = 0.9), race, ASA score, or CCI (3.5 ± 2.37 vs. 3.7 ± 2.63, *p* = 0.5) (Table 4).

### 3.3. Coronal Limb Alignment vs. Femoral Rotation Morphology

There was no statistically significant difference in the mean PCAngle between the varus and valgus group (+2.9°ER ± 1.9° vs. +2.8°ER ± 2.0°, *p* = 0.7). The PCAngle between the sTEA to PCAxis was between 2° to 4° ER in 37% of patients in the valgus group compared to 38% of patients in the varus group (*p* = 0.8). Similarly, the relative rotation of sTEA to PCAxis was 1° to 5° ER in 69% of patients in the valgus group compared to 67% of the patients in the varus group (*p* = 0.8) (Table 4).

There was no statistically significant difference in the relative rotation of sTEA to PCAxis being external (>0°) or internal (<0°) between the two groups (Table 4).

**Figure 3:**
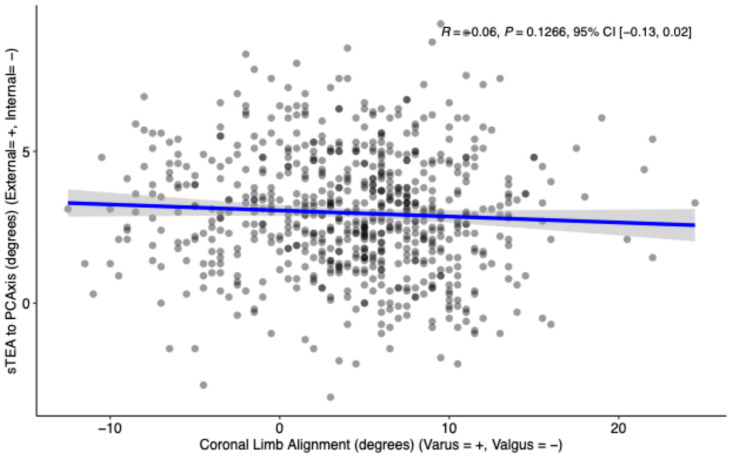
Scatterplot of the coronal limb alignment vs. the relative rotation of sTEA to PCAxis with a linear regression and 95% confidence interval demonstrating a statistically insignificant small negative correlation (R = −0.06, *p* = 0.1266).

Pearson’s product–moment correlation between PCAngle of sTEA to PCAxis and coronal limb alignment was negative, very small, and not statistically significant (r = −0.06, 95% CI [−0.13, 0.02], *p* = 0.1266) (Figure 3).

## 4. Discussion

The purpose of this study was to examine variability in the posterior condylar angle and explore the relationship between the posterior condylar angle and coronal knee alignment. To our knowledge, this is the largest CT-based study to assess the relationship between posterior condylar angle and coronal alignment, offering enhanced generalizability and methodological consistency compared to prior reports relying on intraoperative landmarks or cadaveric measurements in smaller patient cohorts. Our results demonstrate wide variability in the posterior condylar angle (PCAngle) and found no significant relationship with coronal limb alignment. We find that this data underscores the danger of assuming a uniform “3° external” target and highlights the value of patient-specific rotational assessment.

Soft tissue balance has been implicated as one of the most important factors that influence clinical outcomes of TKA [18,19]. An important contributor to satisfactory soft tissue balance is flexion and extension gaps [1,2,10,18,19]. Measured resection techniques use bony landmarks to guide femoral rotation and establish a satisfactory flexion gap [20,21,22,23]. Within the paradigm of measured resection, two of the most commonly used anatomical landmarks in the measured resection technique are the sTEA and PCAxis, with many surgical instruments designed to place femoral implants at 3° external rotation from the PCAxis. Coronal alignment has also been found to have a large effect on flexion and extension spaces and influence on clinical outcomes [21,24]. An important goal of prosthetic knee alignment is to maintain or restore optimal flexion and extension, producing more natural knee function so that patient dissatisfaction and “unnatural knee” perception are reduced [24].

Despite the importance of both coronal alignment and the PCAngle, the relationship between coronal alignment and the size of the PCAngle is not fully understood. Gu et al. first explored the question in a cohort of around 100 osteoarthritic knees measured with intra-operative mechanical jigs and found the PCAngle to be ≈2° larger in valgus limbs than in neutral or varus limbs; however, those values were derived from worn cartilage surfaces and may not reflect contemporary imaging-based techniques [13]. Building on that work, Gu et al. subsequently performed a CT-based computer simulation in 49 knee models and showed that setting the femur in 5–7° valgus frequently produced ≥2 mm mediolateral gap imbalances and altered knee alignment by ≥ 2°, again implying a greater rotational requirement in valgus morphotypes [14]. More recent imaging studies have provided mixed results: Griffin et al. explored the relationship further by looking at the effect of coronal alignment on the rotational alignment of sTEA and found that the PCAngle was over 2° greater in valgus knees than varus or knees with no coronal deformity [25]. Akagi et al. used preoperative CT scans in 111 patients and found that the PCAngle increased if the valgus deformity was over 9° [15]. Luyckx et al. performed a somewhat larger study using preoperative CT scans on 231 patients and also found similar results, reporting that for a 1° change in coronal alignment varus to valgus, they found a 0.1° change in PCAngle between sTEA to PCAxis [16].

While previous results point to a possible relationship between a larger PCAngle and valgus deformity, our results differ and found no significant correlation (R = −0.06, *p* = 0.1266) between the PCAngle and the coronal limb alignment in a dataset of 695 patients (Figure 3). This illustrates that while there may be a small decrease in the PCAngle as the coronal alignment moves further towards varus, it is both statistically and clinically insignificant. The difference in findings may be due to differences in study populations; our larger dataset may give a more generalizable idea of the relationship between coronal alignment and sTEA to PCAxis rotation. It is also likely that the variations in the modalities used to measure landmarks contribute to variation in reported results and confound direct comparison. While some previous studies used preoperative CT scans, others used cadaver measurements, MRI scans, and intraoperative measurements with a posterior referencing guide—all of which often measure off posterior condylar cartilage rather than bone.

Additionally, our study found significant variation in the size of the PCAngle, which has additional implications for surgical alignment strategies. As referenced in the introduction, alignment strategies traditionally use fixed anatomical landmarks (e.g., 3° external to the PCAxis) regardless of pre-operative patient morphology. Our findings here suggest that prioritizing individual ligament balance and joint geometry is likely more reliable than traditional standardized approaches.

Our study does not find coronal alignment to be a reliable metric to guide rotational alignment. This aligns with the principles of functional or kinematic alignment, which traditionally prioritizes individualized joint kinematics over fixed mechanical targets. We therefore suggest that femoral rotation should be derived using other techniques and guided by other patient-specific metrics. We advocate for rotational alignment strategies that incorporate patient-specific anatomical data derived from modalities such as CT-based preoperative planning or functional intraoperative assessments. While not directly analyzed in this study, we hypothesize that this may offer superior reliability over coronal-based estimation alone. We also note that while previous studies used various modalities to measure landmarks, specifically analyzing surgical landmarks using modern 3D CT-based imaging systems provides valuable insights and may enhance decision-making for surgeons using TKA planning software that utilizes 3D bone models derived from CT scans.

The ideal landmark for setting femoral rotation in TKA remains a subject of debate. It is notable that, with the mean PCAngle in our cohort being 3 degrees and the sTEA ranging from 3.1 degrees internal to 9.2 degrees external to the PCAxis, it would have been possible to place the femoral component within 3.2 degrees of the sTEA in all cases while keeping the implant within 0–6 degrees of external rotation from the PCAxis (i.e., within 3 degrees of the common target of 3 degrees of external rotation).

### Limitations

Although larger than prior studies, our sample reflects the demographics of the population served at a large academic medical center in the United States and may not be generalizable to demographically different patient populations. This study also does not address clinical outcomes or PROMs, limiting our ability to make specific clinical recommendations based on these data. The coronal alignment was measured intraoperatively by the robotic software (MAKO v2.0); these angular measurements may have been impacted by flexion contracture and may differ from radiographic measurements. Although the accuracy of the robotic software used has been validated in previous studies, there is certainly the potential for error in the identification of bone landmarks on the CT images. Arthritic knee anatomy can be distorted by osteophytes or subchondral bone irregularities that deform the condylar surfaces. These issues in our dataset also exist in clinical practice, however, making our findings relevant to the clinical scenario.

## 5. Conclusions

In conclusion, our data finds that the sTEA-to-PCAxis relationship, known as the PCAngle, varies from 3 degrees internal to 9 degrees external and cannot be reliably predicted by coronal alignment in mechanically aligned TKA. Our findings suggest that both axes should be independently assessed before determining patient-specific targets for femoral component rotation, therefore limiting unwanted complications such as asymmetric flexion gaps, patellofemoral mal-tracking, and the soft-tissue releases or revisions that may follow malrotation. We recommend incorporating patient-specific 3D imaging assessments of the sTEA and PCAxis to guide rotational alignment. Future work should assess the clinical impact of such personalized strategies on implant longevity and patient-reported outcomes.

## Figures and Tables

**Table 1:** Baseline and demographic variables.

Characteristic	*n* = 695
**Age at Surgery**	
Mean (SD)	67.7 (9.3)
Median (IQR)	68.0 (13.0)
Range	27–94
**Sex**	
Female	486 (69.9%)
Male	209 (30.1%)
**Race**	
White	365 (52.5%)
Black	102 (14.7%)
Asian	49 (7.1%)
Other	179 (25.8%)
**Laterality**	
Left	358 (51.5%)
Right	337 (48.5%)
**Procedure Length (Minutes)**	
Mean (SD)	106.3 (23.9)
Median (IQR)	102.0 (28.0)
Range	58–234
**Body Mass Index**	
Mean (SD)	31.6 (5.1)
Median (IQR)	31.3 (7.1)
Range	20–48
**American Society of Anesthesiologists (ASA) Score**	
1	17 (2.4%)
2	419 (60.3%)
3	258 (37.1%)
4	1 (0.1%)
**Charlson Comorbidity Index (CCI)**	
Mean (SD)	3.6 (2.5)
Median (IQR)	3.0 (3.0)
Range	0–24

**Table 2:** Posterior condylar axis (PCAxis) to surgical transepicondylar axis (sTEA) relative rotation.

Characteristic	*n* = 695
**sTEA to PCAxis (External = +, Internal = −)**	
Mean (SD)	3.0 (2.0)
Median (IQR)	2.9 (2.7)
Range	−3.1 to 9.2
**sTEA to PCAxis between +2 to +4 degrees**	270 (39%)
**sTEA to PCAxis between +1 to +5 degrees**	466 (67%)
**sTEA to PCAxis between +0 to +6 degrees**	595 (86%)
**sTEA to PCAxis > 0 degrees**	647 (93%)
**sTEA to PCAxis < 0 degrees**	43 (6.2%)

**Table 3:** Coronal limb alignment in the cohort.

Characteristic	*n* = 695
**Coronal Limb Alignment (Varus = +, Valgus = −)**	
Mean (SD)	4.3 (5.8)
Median (IQR)	5.0 (7.5)
Range	−12.5 to 24.5
**Varus Alignment > 2 degrees**	465 (67%)
**Valgus Alignment > 2 degrees**	101 (15%)

**Table 4:** Coronal limb alignment vs. demographic variables and femoral rotation morphology.

Characteristic	Valgus > 2 Degrees, *n* = 101 ^1^	Varus > 2 Degrees, *n* = 465 ^1^	*p*-Value
**Sex**			**<0.001** ^2^
Female	85 (84%)	302 (65%)	
Male	16 (16%)	163 (35%)	
**Age at Surgery**			0.9 ^3^
Mean (SD)	67.4 (10.31)	67.9 (8.91)	
Range	41, 94	39, 90	
Median (IQR)	67.5 (11.25)	68.0 (13.00)	
**Race**			0.6 ^2^
White	50 (50%)	246 (53%)	
Black	19 (19%)	63 (14%)	
Asian	6 (5.9%)	32 (6.9%)	
Other	26 (26%)	124 (27%)	
**American Society of Anesthesiologists (ASA) Score**			0.6 ^2^
1	3 (3.0%)	10 (2.2%)	
2	60 (59%)	279 (60%)	
3	38 (38%)	175 (38%)	
4	0 (0%)	1 (0.2%)	
**Body Mass Index**			**0.004** ^3^
Mean (SD)	30.1 (5.39)	31.8 (4.91)	
Range	20, 46	20, 48	
Median (IQR)	30.3 (7.58)	31.5 (7.04)	
**Charlson Comorbidity Index (CCI)**			0.5 ^3^
Mean (SD)	3.5 (2.37)	3.7 (2.63)	
Range	0, 11	0, 24	
Median (IQR)	3.0 (3.00)	3.0 (3.00)	
**Laterality**			0.2 ^2^
Left	46 (46%)	249 (54%)	
Right	55 (54%)	216 (46%)	
**sTEA to PCAxis (External = +, Internal = −)**			0.7 ^3^
Mean (SD)	2.9 (1.90)	2.8 (1.99)	
Range	−3, 7	−3, 9	
Median (IQR)	3.0 (2.80)	2.8 (2.60)	
**sTEA to PCAxis between +2 to +4 degrees**	37 (37%)	179 (38%)	0.8 ^2^
**sTEA to PCAxis between +1 to +5 degrees**	70 (69%)	312 (67%)	0.7 ^2^
**sTEA to PCAxis > 0 degrees**	96 (95%)	427 (92%)	0.3
**sTEA to PCAxis < 0 degrees**	4 (4.0%)	34 (7.3%)	0.2

^1^ n (%); ^2^ Fisher’s exact test; ^3^ Wilcoxon rank sum test.

## Data Availability

The original contributions presented in this study are included in the article/Supplementary Materials. Further inquiries can be directed to the corresponding author.

## Supplementary Materials

The following supporting information can be downloaded at: https://www.mdpi.com/article/10.3390/bioengineering12070727/s1, Table S1: Posterior condylar axis (PCAxis) to surgical transepicondylar axis (sTEA) relative rotation.
