# Supplementary material for: Coronal Alignment Does Not Adequately Predict Femoral Rotation Axes in Total Knee Arthroplasty: Application of a 3D Image-Based Robotic-Assisted Arthroplasty Platform
Source: Bioengineering (Basel). 2025 Jul 1;12(7):727. doi: 10.3390/bioengineering12070727 (PMC12292550; doi:10.3390/bioengineering12070727)
Supplement: Supplementary file 1 [file bioengineering-12-00727-s001.zip › bioengineering-3710087-supplementary.pdf]

Supplementary Material

Table S1. Posterior condylar axis (PCAxis) to surgical transepicondylar axis (sTEA) relative rotation.

Characteristic	N = 695
**sTEA to PCAxis (External= +, Internal = -)**	
Mean (SD)	3.0 (2.0)
Median (IQR)	2.9 (2.7)
Range	-3.1 to 9.2
**sTEA to PCAxis between +2 to +4 degrees**	270 (39%)
**sTEA to PCAxis between +1 to +5 degrees**	466 (67%)
**sTEA to PCAxis between +0 to +6 degrees**	595 (86%)
**sTEA to PCAxis > 0 degrees**	647 (93%)
**sTEA to PCAxis < 0 degrees**	43 (6.2%)
